# The Noises of Great Cities

**Published:** 1895-02-16

**Authors:** 


### THE NOISES OF GREAT CITIES.

Dwellers in London must be thankful for such small mercies as may be vouchsafed them in the way of protection from the misdeeds of their neighbours, and in truth it may be said that such mercies are not great. Mr. Curtis Bennett, the magistrate presiding at the West London Police Court, has recently given warning as to what people must expect if they have the hardihood to live in London. "People," he is reported to have said, "who live in London must put up with noises." A sad fact with which we are all sufficiently familiar. When appealed to by the suffering neighbours of a teacher of music for some mitigation of the nuisance arising from the continued playing of the piano next door, the only suggestion the Court could give was that "people who did not like noise must live out of London." One small crumb of comfort, however, was added for the benefit of those weary ones whose short hours of sleep are broken by the row and racket amid which they have to dwell, for it was said that although the courts would not interfere to stop the playing of the piano or violin up till eleven o'clock at night, "after that time, perhaps, the courts would grant an injunction to stop the playing." This is the possible small mercy for which Londoners must offer thanks, and, in truth, it is a little thing in return for the rents and rates they have to pay. Nuisance is a term of very elastic definition, and it is somewhat curious that offences against the different senses are dealt with so diversely. A smoke, or a smell, or a coarse vibration, such as that caused by electric light works, is dealt with vigorously, but as regards mere noise, musical or otherwise, a man is at the mercy of his neighbours, or of any itinerant ruffians who, under the pretence of selling coals, vegetables, milk, flowers, cat's meat, muffins, and dispensing all the varieties of cacophony which go by the name of street music, rob the residents of their well-earned rest, and impede them in the enjoyment of their properties. These things surely are nuisances even in the daytime, but at night they are certainly "nuisances injurious to health." If people are to stand the turmoil of modern life they must have sleep. On every side we see that want of the sweet restorer is the beginning of nervous breakdown; and, slight as is the hope held out by Mr. Curtis Bennett, and illogical as it may seem to choose eleven as the magic moment when the witching hour of sleep is to commence, it is something to have even this small prospect of a term being put to the nocturnal noises of great cities.

